# Human Papilloma Virus Persistence after Cone Excision in Women with Cervical High Grade Squamous Intraepithelial Lesion: A Prospective Study

**DOI:** 10.1155/2016/3076380

**Published:** 2016-05-12

**Authors:** Laurențiu Pirtea, Dorin Grigoraş, Petru Matusz, Marilena Pirtea, Lavinia Moleriu, Anca Tudor, Răzvan Ilina, Cristina Secoşan, Florin Horhat, Octavian Mazilu

**Affiliations:** ^1^Department of Obstetrics and Gynecology, University of Medicine and Pharmacy “Victor Babeş”, Timişoara, 300041 Timiș, Romania; ^2^Department of Anatomy, University of Medicine and Pharmacy “Victor Babeş”, Timişoara, 300041 Timiș, Romania; ^3^Department of Obstetrics and Gynecology, County Hospital Timişoara, 300202 Timiș, Romania; ^4^Department of Informatics and Statistics, University of Medicine and Pharmacy “Victor Babeş”, Timişoara, 300041 Timiș, Romania; ^5^Department of Surgery, University of Medicine and Pharmacy “Victor Babeş”, Timişoara, 300041 Timiș, Romania; ^6^Department of Microbiology, University of Medicine and Pharmacy “Victor Babeş”, Timişoara, Piata Eftimie Murgu, No. 1, 300041 Timiș, Romania

## Abstract

*Background*. Persistent human papillomavirus (HPV) infection is a necessary event in cervical cancer tumorigenesis. Our objectives were to estimate the rate of HPV infection persistence after large loop excision of the transformation zone (LEEP) in patients with high grade squamous intraepithelial lesions (HSIL) and to investigate if HPV persistence is type related.* Methods*. We conducted a prospective study on 89 patients with HSIL treated with LEEP. DNA HPV was performed before surgery and at 6, 12, and 18 months after LEEP.* Results*. Four patients were excluded from the study. The HPV persistence in the remaining 85 patients was 32.95% (6 months), 14.12% (12 months), and 10.59% (18 months). Type 16 had the highest persistence rate, 23.5% (6 months), 11.8% (12 months), and 8.2% (18 months). Coinfection was found to be 54.12% before LEEP and 18.8% (6 months), 4.7% (12 months), and 3.5% (18 months) after LEEP. The rate of coinfections including type 16 was 46.83% of all coinfections. Coinfection including type 16 was not correlated with higher persistence rate compared to infection with type 16 only.* Conclusions*. HPV infection is not completely eradicated by LEEP in patients with HSIL lesion on PAP smear. HPV persistence after LEEP is influenced by HPV type. HPV type 16 has the highest persistence rate.

## 1. Introduction

Persistent human papillomavirus (HPV) infection is a necessary event in cervical cancer tumorigenesis. Virtually all tumor cells in a cervical cancer contain sequences of HPV [1]. HPV is the most frequent sexually transmitted disease in the world. The majority of infections are transient, up to 70% regressing in the first year and up to 90% in 2 years; 10–20% of the infections persist, allowing the evolution of preneoplastic lesions to cancer [2]. Only 40 of the 200 known HPV genotypes present tropism for the anogenital mucosa and 18 of those 40 types are directly related to cervical cancer [3, 4]. Fifteen HPV types have been defined as high risk-human papilloma virus (HR-HPV) types with strong oncogenic potential: 16, 18, 31, 33, 35, 39, 45, 51, 52, 56, 58, 59, 68, 73, and 82. These HR-HPV types account for 95% of all cervical cancers. Simultaneous infections with multiple HPV types are common [5].

HPV 16 and 18 are the most common HR-HPV types worldwide and account for about 70% of all squamous cells carcinomas (SCC) and for up to 85% of all adenocarcinomas. HPV 16 is the most carcinogenic HPV genotype and HPV 18 causes a greater proportion of glandular cancers compared to squamous cell carcinoma. After HPV 16 and 18, the six most prevalent types that account for an additional 20% are types 31, 33, 35, 45, 52, and 58 [6].

Cone excision of the uterine cervix such as large loop excision of the transformation zone (LEEP) is not only a diagnostic procedure but also an appropriate treatment for cervical intraepithelial neoplasia (CIN) [7, 8]. However, CIN can recur, and invasive cervical carcinoma can develop, following such CIN treatment. There is increasing evidence that testing for the presence of high risk-human papilloma virus (HR-HPV) after LEEP may help predict the likelihood of persistent or recurrent disease [9, 10].

The objectives of our study were to estimate the rate of HPV infection persistence after LEEP in patients with high grade squamous intraepithelial lesions (HSIL) and to investigate if HPV persistence is type related.

## 2. Materials and Methods

*Patient Selection.* We performed a prospective study. We included in the study all patients with HSIL cytology on PAP smear who were referred for LEEP to the Department of Obstetrics and Gynecology of the University of Medicine and Pharmacy “Victor Babeş”, Timişoara, between January 2010 and May 2014. Conventional cytology was performed and evaluated according to the criteria of Bethesda 2001. All patients were evaluated by colposcopy and the criteria of the International Federation for Cervical Pathology and Colposcopy (IFCPC) were used. All patients underwent LEEP under colposcopic vision after application of Lugol solution. During the procedure, all colposcopically abnormal findings were excised, aiming for a tissue depth of at least 6 mm. All procedures were performed by the same team of surgeons. DNA HPV testing was performed before LEEP in all cases. DNA HPV testing was repeated at 6, 12, and 18 months after LEEP. Patients who were negative for DNA HPV before LEEP were excluded from the study. All samples were examined using LINEAR ARRAY HPV Genotyping Test (CE-IVD), based on reverse hybridization of amplicons. The DNA of 37 HPV types (6, 11, 16, 18, 26, 31, 33, 35, 39, 40, 42, 45, 51, 52, 53, 54, 55, 56, 58, 59, 61, 62, 64, 66, 67, 68, 69, 70, 71, 72, 73, 81, 82, 83, 84, IS39, and CP6108) was detected in cervical samples by multiplex PCR targeted to the conserved L1 region of the viral genome. The Gene Amp PCR System 9700 was used for genotyping test according to the manufacturer's instructions. Automated hybridization and detection of HPV DNA was done on ProfiBlot 48 (Tecan Trading AG, Zurich, Switzerland).

All specimens were sent for histopathological exam. Patients with positive resection margins after LEEP were excluded from the study.

Informed consent was obtained from all patients prior to their inclusion in the study. All procedures have been performed in accordance with the ethical standards laid down in the 1964 Declaration of Helsinki and its later amendments and were approved by the Institutional Review Board and Ethical Committee of University of Medicine and Pharmacy “Victor Babeş”, Timişoara.

Statistical analysis was performed using SPSS v17 and Epi Info 7. For computing *p* values we used nonparametric tests (Wilcoxon sign rank and chi square).

## 3. Results

A total of 89 patients were referred to our clinic with HSIL on PAP smear test. After the first DNA HPV testing, 2 patients were found negative and were excluded from the study. We excluded those patients because we wanted to investigate HPV persistence after LEEP. Since there was no infection before LEEP, we excluded the patients from the trial.

Another 2 patients had positive margins on the LEEP specimen and were also excluded from the study. The remaining 85 patients were tested for DNA HPV at 6, 12, and 18 months after LEEP. All remaining patients were positive for HR-HPV before LEEP. The HPV types detected before LEEP were 50.6% type 16, 24.7% type 18, 20% type 31, 24.7% type 33, 11.8% type 35, 7.1% type 45, 27.1% type 52, 9.4% type 58, 9.4% type 6, 5.9% type 11, and 5.9% other types. At 6 months after LEEP, the overall persistence was 32.95% (28 patients), at 12 months 14.12% (12 patients), and at 18 months 10.59% (9 patients). The rate of persistence in our group at 6, 12, and 18 months for each HPV type is shown in Table 1. Viral persistence at 12 and 18 months was observed only in patients positive at 6 months. Type 16 was found to be with the highest persistence rate, 23.5% at 6 months, 10.6% at 12 months, and 8.2% at 18 months. Type 16 was associated with significantly increased risk of persistence compared with the other high risk types (Table 2).

Coinfection, defined as the presence of more than one HPV type, was found to be 54.12% before LEEP, 18.8% at 6 months, 4.7% at 12 months, and 3.5% at 18 months after LEEP (Table 1). Coinfections including type 16 represented 46.83% of all coinfections. Patients positive for type 16 were found to have significantly higher risk of infection persistence than patients presenting infections or coinfections with other types (Tables 3 and 4). Coinfection including type 16 was not correlated with higher persistence rate than infection with type 16 only (Table 5).

## 4. Discussion 

Cervical cancer remains a leading cause of morbidity and mortality in women worldwide [11, 12]. It is caused by the acquisition and persistence of types of high risk-human papilloma virus (HR-HPV) infection and the subsequent malignant transformation of cervical epithelial cells [13]. Persistent HPV infection is a major factor in CIN development. The natural history of the progression of HPV infection to cervical lesion or clearance was investigated by Jaisamrarn et al. 2013. They found that overall 53%, 79%, 87%, and 89% of all HPV infections were cleared at 12, 24, 36, and 48 months, respectively, and that HPV 16 and HPV 31 were significantly less likely to clear than a nononcogenic HPV [14]. The persistence of high risk HPV infection is a key factor for the development of cervical cancer [15, 16], and detection of viral persistence can be used to identify the women with the greatest risk of cervical cancer [17].

Cone excision of the cervix is considered both diagnostic and therapeutic procedure that can effectively eradicate HR-HPV infection and CIN. Despite the removal of the entire lesion by cone excision with negative margins, the HPV infection can persist in some cases. Studies investigating the clearance/persistence of HPV infection after LEEP have reported that age, lesion grade, and margin status are risk factors for HPV persistence. Our persistence rate at 6 months was 32.95%. Other authors, using the same procedure, reported lower rates of HPV persistence at 6 months, ranging from 14.3% to 21.5% [18–21]. We consider that the selection of only patients with HSIL and the fact that all patients in our study had HR-HPV infections are responsible for our higher persistence rate. Park et al. and Nam et al. also found that high grade lesions are risk factors for HPV persistence after LEEP [19, 22].

We excluded patients with positive margins after resection from our study because we wanted to investigate the persistence of HPV infection in patients with negative margins. The presence of positive margins is considered a major factor for HPV persistence and disease recurrence and progression. Alonso et al. 2006 found that positive cone margins were significantly associated with higher risk of recurrence [23]. We selected patients with HSIL because they are more likely to have HPV infection with high risk types, and HSIL lesions are more likely to progress to invasive disease. We excluded patients with negative HPV DNA before LEEP because our goal was to investigate HPV infection persistence after LEEP.

Several authors investigated and found that viral load prior to LEEP is a risk factor for HPV persistence [19, 22, 24]. High viral loads, RLU/PC ≥ 100, were considered risk factors for HPV persistence and disease recurrence [20, 22]. Since the measurement of HPV viral load is not widely available, we investigated if the risk of HPV persistence after LEEP is related to certain types of HPV. We consider this helpful for risk stratification and the selection of the group of patients that should be evaluated more carefully. We focused on type related persistence, because DNA HPV is a more widely used test than HPV viral load evaluation.

The identification of the same HPV type before and after LEEP was considered persistence. We did not find new types of HPV after LEEP in any patient. Viral persistence at 12 and 18 months was found only in patients positive at 6 months after LEEP. We consider that the probability of reinfection during the study is low, although it is difficult to completely exclude it.

In our study HPV type 16 was found to be a factor that favors HPV persistence after LEEP. Our results are in agreement with Nam et al. 2009 [22] who investigated the factors associated with HPV persistence after conization in a similar group (77 patients) and found that preoperative HPV type 16 infection was the only significant independent factor (*p* = 0.021) for HPV persistence out of age, cytology, punch biopsy histology, HPV viral load, and conization histology.

Our results also indicate that most HPV infections are cleared at 12 months after surgery, and very few are cleared after this interval. We suggest that the HPV DNA follow-up endpoint after LEEP should be 12 months. We also consider that patients with infection persistence after 12 months are more likely to develop disease progression than infection clearance, but more data are necessary in order to prove this statement. We suggest that HPV DNA at 12 months after LEEP could be a useful tool, in order to identify the patients that are likely to experience disease progression.

Persistence or clearance of HPV DNA is considered an early valid prognostic marker of failure or cure after treatment for cervical dysplasia and is more accurate than cytology or section margin status at the time of LEEP [25]. According to the meta-analysis performed by Kocken et al. 2012 HPV test should be included in posttreatment testing 6 months after treatment, because it has a higher sensitivity than cytology in detecting high grade posttreatment disease and has a similar specificity [26]. The recurrence of cervical dysplasia after LEEP is also related to HPV persistence. Several investigators have analyzed the sensitivity and specificity of HPV DNA testing compared with follow-up cytology to more accurately detect residual/recurrent disease after treatment [27, 28]. HPV testing was found to be more sensitive than follow-up cytology, with comparable specificity [24, 28]. Women who are HPV positive after surgery are at higher risk for treatment failure [24, 29, 30].

We noticed the persistence of types 6 and 11 at 12 months. This was also reported by Brismar et al. Those are not considered high risk types, but they are associated with condyloma [31].

The interest regarding coinfection with multiple types of HPV has increased in the past years, along with the possibility of vaccination and also due to the discovery that the immune response seems to be type specific [32].

The incidence of coinfection reported in literature is variable, ranging from 19% to up to 43.2%. This is due to factors such as age, sexual behavior, and immune response and HPV detection method. Our coinfection rate was 54.12%, and the rate of coinfections including type 16 was 46.83% of all coinfections [32, 33].

On a study on 1124 patients, Liaw et al. found that HPV 16 had persisted longer than other types, but it did not alter subsequent persistence of other concomitant HPV infections. This concurs with our results that type 16 has the highest persistence rate (Table 2). We also found that infection with type 16 has higher persistence rate than coinfection without type 16 (Table 3). The coinfection including type 16 was not correlated with higher persistence rate compared to infection with type 16 alone (Table 5). This indicates that the presence of type 16 is the most important factor for infection persistence [32, 34].

The strengths of study are represented by the prospective nature of the study and the fact that only patients with HSIL were selected. This way we investigated the very category of patients that are likely to be infected with HR-HPV and that are exposed to recurrence after LEEP and disease progression to cancer.

## 5. Conclusions 

HPV infection is not completely eradicated by LEEP in patients with HSIL lesion on PAP smear. HPV persistence after LEEP is influenced by HPV type. HPV type 16 has the highest persistence rate.

Our results strongly advocate introducing the HPV DNA test in the follow-up of the patients that underwent LEEP for HSIL, especially if HPV type 16 was identified prior to LEEP.

## Supplementary Material

The data used for the statistical analysis regarding HPV infection with the tested HPV-DNA, determined before LEEP (1 = present, 0 = absent), and at 6, 12, and 18 months after LEEP, and also the age of the patients investigated.

## Figures and Tables

**Table 1:** The rate of persistence at 6, 12, and 18 months for each HPV type computed for all 85 subjects.

HPV types	Before conization	6 months	12 months	18 months
16	43 (50.6%)	20 (23.5%)	9 (10.6%)	7 (8.2%)
52	23 (27.1%)	5 (5.9%)	0 (0%)	0 (0%)
18	21 (24.7%)	4 (4.7%)	1 (1.2%)	0 (0%)
33	21 (24.7%)	2 (2.4%)	0 (0%)	0 (0%)
31	17 (20%)	1 (1.2%)	0 (0%)	0 (0%)
35	10 (11.8%)	0 (0%)	0 (0%)	0 (0%)
58	8 (9.4%)	1 (1.2%)	0 (0%)	0 (0%)
6	8 (9.4%)	2 (2.4%)	2 (2.4%)	2 (2.4%)
45	6 (7.1%)	0 (0%)	0 (0%)	0 (0%)
11	5 (5.9%)	1 (1.2%)	1 (1.2%)	1 (1.2%)
Other types	5 (5.9%)	0 (0%)	0 (0%)	0 (0%)
Coinfections	46 (54.12%)	16 (18.8%)	4 (4.7%)	3 (3.5%)

**Table 2:** The *p* value obtained by comparing HPV 16 to the other HPV types using *χ* ^2^ test.

*p* value, obtained after applying a chi square test				
HPV types	Before conization	6 months	12 months	18 months
16 versus 18	<0.001^s^	<0.001^s^	0.023^s^	0.02^s^
16 versus 31	<0.001^s^	<0.001^s^	0.006^s^	0.02^s^
16 versus 33	<0.001^s^	<0.001^s^	0.006^s^	0.02^s^
16 versus 35	<0.001^s^	<0.001^s^	0.006^s^	0.02^s^
16 versus 45	<0.001^s^	<0.001^s^	0.006^s^	0.02^s^
16 versus 52	0.003^s^	0.002^s^	0.006^s^	0.02^s^
16 versus 58	<0.001^s^	<0.001^s^	0.006^s^	0.02^s^
16 versus 6	<0.001^s^	<0.001^s^	0.063^ns^	0.178^ns^
16 versus 11	<0.001^s^	<0.001^s^	0.023^s^	0.073^ns^
16 versus other types	<0.001^s^	<0.001^s^	0.006^s^	0.02^s^

^s^Significant differences (with 0.05; 0.01; and 0.001 level of significance).

^ns^Insignificant differences (with 0.05 level of significance).

From the second table we can see that the most frequent HPV type is 16. More than that, this HPV type seems to be the most persistent and dangerous. We can see that there are extremely significant differences between HPV 16 and the other HPV types, in most of the cases.

**Table 3:** The *p* value obtained using *χ* ^2^ test, comparing patients positive for HPV type 16 and patients having coinfections (without HPV 16). HPV 16 was correlated with significantly higher risk of persistence (*p* < 0.5) at 6 and 12 months.

	Before conization	6 months	12 months	18 months
*p* value	*p* = 0.87	*p* = 0.01	*p* = 0.02	*p* = 0.09
RR	RR = 1.02	RR = 2.5	RR = 4.5	RR = 3.45
95% interval of confidence	RR ∈ (0.75; 1.38)	RR ∈ (1.16; 5.3)	RR ∈ (1; 20.21)	RR ∈ (0.73; 16.17)

**Table 4:** The *p* value obtained using *χ* ^2^ test, comparing coinfections including HPV 16 + other HPV types, with coinfections without HPV 16. Coinfection including type 16 was correlated with higher persistence rate than coinfections without type 16.

	Before conization	6 months	12 months	18 months
*p* value	*p* = 0.01	*p* = 0.03	*p* = 0.05	*p* = 0.07
RR	RR = 1.07	RR = 1.75	RR = 2.75	RR = 3
95% interval of confidence	RR ∈ (1.01; 1.14)	RR ∈ (1.02; 2.99)	RR ∈ (0.91; 8.29)	RR ∈ (0.84; 10.69)

**Table 5:** The *p* value obtained using *χ* ^2^ test, comparing coinfections with HPV 16 and other HPV types and only HPV 16. Coinfection including type 16 was not correlated with higher persistence rate than infection with type 16 alone.

	Before conization	6 months	12 months	18 months
*p* value	*p* = 0.36	*p* = 0.01	*p* = 0.02	*p* = 0.07
RR	RR = 0.86	RR = 0.4	RR = 0.22	RR = 0.14
95% interval of confidence	RR ∈ (0.62; 1.19)	RR ∈ (0.19; 0.86)	RR ∈ (0.04; 0.99)	RR ∈ (0.01; 1.13)

## Additional Points

Name of register is Laurențiu Pirtea.
